# Can Your DNA Influence Your Bet-Placing? The Impact of Cannabinoid Receptor 1 Gene on Gambling Tasks

**DOI:** 10.3389/fnhum.2018.00458

**Published:** 2018-11-27

**Authors:** Huihui Qin, Jianmin Zeng, Hong Chen, Ling Deng, Li Su

**Affiliations:** ^1^Sino-Britain Centre for Cognition and Ageing Research, Faculty of Psychology, Southwest University, Chongqing, China; ^2^Department of Neurosurgery, The Ninth People’s Hospital of Chongqing, Chongqing, China; ^3^Department of Psychiatry, University of Cambridge, Cambridge, United Kingdom

**Keywords:** behavioral economics, neuroeconomics, behavioral genetics of decision-making, decision-making under risk, cognitive neuroscience

## Abstract

Are we placing a bet by ourselves or has our DNA already made the decision for us? Previous research has suggested that some genes related to dopamine or serotonin can influence our non-bet-placing decision-making, but little is known about whether cannabinoid-related genes can impact how much people bet. To investigate this issue, we focused on rs1049353, a single-nucleotide polymorphism of the cannabinoid receptor 1 (*CNR1*), because it is related to addictive behavior and reward processing. In this study (*N* = 377), we used a modified Cambridge gambling task to test the effect of *rs1049353* polymorphism on how much people bet. We found that participants who are homozygous for C allele placed significantly larger bets than C/T carriers [*F*(1,371) = 7.805, *P* = 0.005]. We further studied the gene expression map in human brains and found that the *CNR1* gene is overexpressed in striatum, amygdala, and hippocampus. These brain structures are known to underpin reward and risk processing. Our findings suggest that, to some extent, high-level social decision-making even like bet-placing could be influenced by a single genetic locus variation in healthy volunteers. In addition, such effects were likely to be mediated by key brain regions in the reward- and risk-processing networks.

## Introduction

People’s decision-making under risk often shares a common tendency but there also exist substantial individual differences. This variability can come from many sources, including environment, individual personality traits, and differences in brain structure and function. A number of studies have revealed heterogeneity in individuals’ risk tolerance and risk-taking (Kahneman and Tversky, 1992; Barsky et al., 1997; Anderson et al., 2015). Previous research has explained individual differences from a demographic perspective, such as sex (Barsky et al., 1997; Croson and Gneezy, 2009), age (Barsky et al., 1997; Mather et al., 2012), race (Barsky et al., 1997; Blum et al., 1999), religion (Barsky et al., 1997; Leon and Pfeifer, 2017), education, income (Donkers et al., 2001), and so on. However, these variables explained only some of the variance. Existing twin-studies showed that individual differences can also be associated with genetic factors, which account for an estimated 20–57% variance of individual differences in risk-taking behavior (Anokhin et al., 2009; Cesarini et al., 2009; Zhong et al., 2009). Animal studies also showed that decision-making under risk could be moderated by genetic difference in rats (Jentsch et al., 2010; Ashenhurst et al., 2012).

Regarding the neurobiological basis of decision-making, the striatum is the terminal region of the dopaminergic mesolimbic system, which is involved in motivational processes (Di et al., 2004; Everitt and Robbins, 2005). In addition, many animal and human studies have identified the ventral striatum playing a crucial role in reward processing (Parkinson et al., 2000; Schultz et al., 2000; Mobbs et al., 2003). During reward processing, increased ventral striatum activity was associated with dopamine release in this brain region (Knutson and Gibbs, 2007; Schott et al., 2008), and the level of striatal dopamine was associated with the function of the ventral hippocampus (Grace, 2016). For instance, stimulating the ventral hippocampus can increase the concentration of dopamine strongly (Blaha et al., 2010).

Clinical evidence also demonstrated that the basolateral amygdala was involved in risk-based decision-making (Floresco et al., 2008; Gowin et al., 2013). Patients with damage to the basolateral amygdala tended to make more risky choices in risky decision tasks (Bechara et al., 1999; Orsini et al., 2015). Reduced amygdala activation has been found to diminish the framing effect (De Martino et al., 2010).

In genetic studies, it has been shown that rs1049353 polymorphism of the cannabinoid receptor 1 (*CNR1*) gene affects addictive behaviors such as alcohol, marijuana, and heroin dependence (Hartman et al., 2009; Proudnikov et al., 2010; Agrawal et al., 2012; Yang et al., 2014). *Rs1049353* is located in exon4 of the *CNR1* gene. The *CNR1* gene is located on human chromosome 6 and encodes the cannabinoid receptor 1 (Mackie, 2008; Onaivi, 2009). Specifically, the C/C genotype of *rs1049353* was a risk factor for drug dependence (Schmidt et al., 2002; Hartman et al., 2009; Proudnikov et al., 2010; Yang et al., 2014). Drug abuse was usually associated with risky behavior, and disorders associated with substance use have been related to the personality trait of impulsivity (Ehlers et al., 2007). Adults with a history of drug dependence also chose risky options more often (Lane and Cherek, 2000) and increased risky decision-making in the Iowa gambling task (Lane et al., 2005). Based on the above, we hypothesized that rs1049353 polymorphism of the cannabinoid receptor 1 gene would affect risk- and reward-related bet placing in our experiment because subjects with the C/C genotype in rs1049353 are more susceptible to drug dependence, which directly involves risk- and reward- processing.

In this study, we determined the subjects’ genetic information for the CNR1 gene and modified the Cambridge gambling task (Rogers et al., 1999) to measure their risk behavior and reward processing. In addition, we obtained the gene expression map from the Allen Human Brain Atlas (Hawrylycz et al., 2012) to further investigate the neurobiological mechanisms linking the CNR1 genotype with behavior performance in our experiment.

## Materials and Methods

### Participants

Totally 377 Han Chinese undergraduate students (age mean ± sd = 19.75 ± 1.02) both finished the experimental task and provided valid saliva samples for genotyping. The study was approved by the local Ethics Committee. All participants provided written consent.

### Experimental Design

**FIGURE 1:**
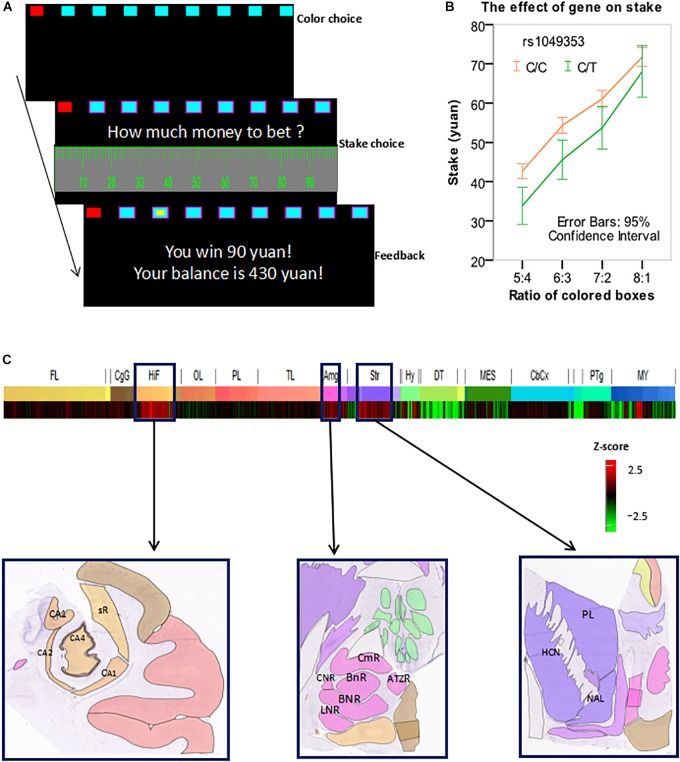
**(A)** An exemplar trial of the modified Cambridge gambling task (MCGT). **(B)** Stake was influenced by both box ratio and genotype. **(C**) The CNR1 gene was overexpressed in the hippocampus, amygdala, and striatum. Z-score bar: Higher redness represents a higher expression level of the CNR1 gene in a brain region. HIF, hippocampal formation; CA1, CA1 field, right; CA2, CA2 field, right; CA4, CA4 field, right; sR, subiculum, right. AMG, amygdala; CmR, cortico-medial group, right; BnR, basomedial nucleus, right; BNR, basolateral nucleus, right; LNR, lateral nucleus, right; CNR, central nucleus, right. Str, striatum. HCN, head of caudate nucleus, left; PL, putamen, left; NAL, nucleus accumbens, left. Image credit for sub-figures in **(C)**: Allen Institute.

In this study, we modified the Cambridge gambling task (MCGT), as shown in Figure 1A. In each trial, an array of nine red and blue boxes were displayed on the screen. Each subject was asked to guess which color of boxes contained a yellow token. The box in which the token was hidden was randomly allocated with a uniform distribution. The ratio between the numbers of red and blue boxes varied from 1:8 to 8:1 and red boxes were always placed on the left-hand side. The subjects chose a color of boxes by pressing a corresponding key with the middle finger (red) and index finger (blue) of their left hands. The boxes of the chosen color were then marked with pink borders. The subjects then needed to decide how much money to bet on their choice.

In each trial, a subject could bet from 0 to 100 Chinese yuan (equivalent to about 15 USDs) by clicking a corresponding mark on the scale with the mouse (say X yuan). A yellow token would then randomly appear in one of the nine boxes. If the subject’s chosen color was the same as the color of the box in which the token was hidden, then she won X yuan, which would be added into the total balance; otherwise, she lost X yuan, which would be subtracted from the balance. The goal of the subject was to maximize the total balance. Before the actual tests, there were two exercise trials. The actual experiment contained 72 trials presented in a randomized order. To avoid the order effect on individual differences, all subjects’ experiments had the same order. There was a break halfway through the task.

### Genotyping

For *rs1049353*, the genotypes were determined by the MassARRAY system (Agena iPLEX assay, San Diego, CA, United States). First, we isolated approximately 10–20 ng of genomic DNA from the saliva. The polymerize chain reaction (PCR) primers used in this study were ACGTTGGATGAATGCAGCCAGTGTTCACAG and ACGTTGGATGACAGACATGGTTACCTTGGC. The sample DNA was amplified by a multiplex PCR reaction, and the obtained products were used for locus-specific single-base extension reaction. Unextended primers used in this study were ACCTTGGCAATCTTGAC. At last, the resulting products were desalted and transferred to a 384-element spectral array. The alleles were discriminated by mass spectrometry (Agena, San Diego, CA, United States). The *rs1049353* genotype was coded as a categorical variable (C/C, C/T, and T/T) for subsequent analysis.

### Gene Expression Map

The gene expression map was obtained from the Allen Human Brain Atlas^1^ by searching for brain regions that have overexpression of CNR1. Details of gene expression analysis methods can be found on the website.

## Results

### The MCGT Task

First, we averaged stakes within the subjects to obtain within-subject mean, based on which we calculated between-377-subject mean and standard error. The resulting mean was 56.42, and the standard error was 0.90. Using a similar calculation for each ratio respectively, we obtained more special descriptive results, as shown in Table 1.

**Table 1:** Descriptives.

Ratio	Stake	
	Mean	Std. Error
5:4	41.43	0.91
6:3	53.06	0.95
7:2	60.04	0.99
8:1	71.16	1.14


### Genotype Equilibrium

Out of the 377 DNA samples, for rs1049353, 319 subjects were C allele homozygote (C/C), 54 were heterozygote (C/T), and four were homozygous for the T allele (T/T). The distribution was consistent with the Hardy–Weinberg equilibrium [X^2^(1) = 0.98, *p* = 0.61]. The allele frequencies in the male samples (C/C = 93, C/T = 16, T/T = 2) showed no deviation from the Hardy–Weinberg equilibrium [X^2^(1) = 1.64, *p* = 0.41]. In the females, 226 were C homozygotes, 38 were heterozygotes, two were T homozygotes, consistent with the Hardy–Weinberg equilibrium [X^2^(1) = 0.08, *p* = 0.96]. In the following analysis, the genotypes of rs1049353 were dichotomized. The number of the T/T genotype (*N* = 4) is extremely small in the sample. If we include this genotype in the analysis, large random errors may occur. Therefore, we excluded the subjects with the T/T genotype (*N* = 4) from subsequent analysis.

### The Effect of *rs1049353* Polymorphism on Stake

In this analysis, we used only the trials wherein a subject made a correct and advantageous choice in the 1st step, i.e., she chose the color that was painted on more boxes. The percentage of these trials was 91.8%. We performed a 2 × 4 mixed effect ANOVA with within-subject factor (box ratio) and between-subject factor (genotype) to test the effect of rs1049353 polymorphism on stakes. There was a significant main effect of box ratio [*F*(3,1113) = 345.130, *p* < 0.0001]. The main effect of gene polymorphism was also significant [*F*(1,371) = 7.805, *p* = 0.005]. No significant interaction [*F*(3,1113) = 2.822, *p* > 0.05] was found. Further analysis showed that subjects with the C/C genotype placed significantly higher stakes than C/T carriers at all ratios except 8:1 (see Figure 1B).

### CNR1 Gene Expression Map

As previously explained, we used the Allen Human Brain Atlas and found that the CNR1 gene was overexpressed in three brain regions: hippocampus, amygdala, and striatum (see Figure 1C).

## Discussion

Previous studies have explored the relationship between gene variation and decision-making but mostly focused on genes related to the serotonin and dopamine systems and non-bet-placing decision-making (Kang et al., 2010; Stoltenberg et al., 2011; Mayseless et al., 2013). This article reports the impact of the gene of the cannabinoid system on decision-making related to bet-placing.

In this study, *rs1049353* polymorphism was found to significantly influence performance in the MCGT. Subjects homozygous for the C allele placed higher stakes than the C/T carriers in the task. These results were consistent with previous findings that subjects with such genotype were more susceptible to drug addiction and risk-taking behavior. Using the Allen Human Brain Atlas, we found that the CNR1 gene was overexpressed in three brain regions: striatum, hippocampus, and amygdala. These brain regions are important centers in the risk- and reward-processing networks in the brain and have been shown to be critical in decision-making, as previously discussed in Introduction. Therefore, this neural network is likely to play a significant role in mediating the effect of CNR1 on risk- and reward-related bet-placing behavior in our data.

Our findings have implications on understanding neurobiological and genetic factors in high-level and complex social decision-making and clinical conditions related to cannabinoid systems. However, the precise molecular and brain mechanism by which this process occurs awaits future research.

## Ethics Statement

The protocol was approved by the Administrative Committee of Psychological Research at Southwest University. All subjects gave written informed consent in accordance with the Declaration of Helsinki.

## Author Contributions

All authors conceived and designed the experiments, collected and analyzed the data, and wrote and revised the manuscript.

## Conflict of Interest Statement

The authors declare that the research was conducted in the absence of any commercial or financial relationships that could be construed as a potential conflict of interest.

## Footnotes

1 http://human.brain-map.org/
